# Robust Powerline Equipment Inspection System Based on a Convolutional Neural Network

**DOI:** 10.3390/s18113837

**Published:** 2018-11-08

**Authors:** Zahid Ali Siddiqui, Unsang Park, Sang-Woong Lee, Nam-Joon Jung, Minhee Choi, Chanuk Lim, Jang-Hun Seo

**Affiliations:** 1 Department of Computer Science & Engineering, Sogang University, 35 Baekbeom-ro, Mapo-gu, Seoul 04107, Korea; zahid@sogang.ac.kr (Z.A.S.); unsangpark@sogang.ac.kr (U.P.); 2 Department of Electronic Engineering, NED University of Engineering & Technology, Karachi-75270, Pakistan; szahid@neduet.edu.pk; 3 Department of Software, Gachon University, 1342 Seongnamdae-ro, Sujeong-gu, Seongnam 13120, Korea; 4 Smart Distribution Lab. SW Platform Center, KEPCO Research Institute, 105 Moonji-ro, Daejeon 34056, Korea; namjoon.jung@kepco.co.kr (N.-J.J.); minhee.choi@kepco.co.kr (M.C.); chanuk_lim@kepco.co.kr (C.L.); 5 ST Vision, 35 Baekbeom-ro, Mapo-gu, Seoul 04107, Korea; janghun.seo@stvision.net

**Keywords:** convolutional neural networks, deep learning, powerline equipment, insulators, cut-out-switches, computer vision, defect analysis, gunshot damage, ellipse detection, electrical safety

## Abstract

Electric power line equipment such as insulators, cut-out-switches, and lightning-arresters play important roles in ensuring a safe and uninterrupted power supply. Unfortunately, their continuous exposure to rugged environmental conditions may cause physical or electrical defects in them which may lead to the failure to the electrical system. In this paper, we present an automatic real-time electrical equipment detection and defect analysis system. Unlike previous handcrafted feature-based approaches, the proposed system utilizes a Convolutional Neural Network (CNN)-based equipment detection framework, making it possible to detect 17 different types of powerline insulators in a highly cluttered environment. We also propose a novel rotation normalization and ellipse detection method that play vital roles in the defect analysis process. Finally, we present a novel defect analyzer that is capable of detecting gunshot defects occurring in electrical equipment. The proposed system uses two cameras; a low-resolution camera that detects insulators from long-shot images, and a high-resolution camera which captures close-shot images of the equipment at high-resolution that helps for effective defect analysis. We demonstrate the performances of the proposed real-time equipment detection with up to 93% recall with 92% precision, and defect analysis system with up to 98% accuracy, on a large evaluation dataset. Experimental results show that the proposed system achieves state-of-the-art performance in automatic powerline equipment inspection.

## 1. Introduction

Powerline equipment such as insulators, cut-out-switches, and lightning arresters plays vital roles, on either the power generation or the distribution side, for the safe delivery of electricity to the end user [1]. Since these equipment has to bear severe weather conditions, high mechanical tension, and extreme voltage power, they are easily damaged, in which case they must be replaced or repaired before their electrical life end [2]. Due to the uncertainty of the life of these insulators, electric companies must take preventive measures to monitor these insulators. Traditional monitoring methods require electric repairmen to climb the pole and visually or electrically analyze the defects, which is time-consuming, labor-intensive and dangerous. Nowadays, some electric companies have started using patrolling helicopters for high power lines in remote areas and ground vehicles in urban areas to take high-resolution pictures or videos of the power lines, poles, and insulators, and then analyze them for their potential defects [3].

Recent studies and advancements in the field of image processing and computer vision, especially the development of Convolutional Neural Networks (CNN) have enabled improving the performance of image analysis at the level of near human intelligence. On the other hand, with the rapidly expanding scale of power grids, traditional methods of manual inspection using electromagnetic field meters is far from satisfying the growing demand, due to the vulnerability to bad weather conditions and high prices [4,5,6]. Consequently, in recent years, many researchers have developed image processing techniques to detect electrical facilities including power lines [7,8,9,10], electric poles [11,12,13] and insulators [14,15,16,17] and analyze their defects [18] using aerial or ground images. Based on the availability of a large database of electromagnetic signals emitted by defected and healthy insulators, automatic signal classification using neural networks [19,20] can also help to detect defective electrical equipment.

Zhang et al. [17] presented a simple method to detect insulators from aerial images by binarizing the intensity image and then applying morphological operations. However, the detection is limited to tempered glass insulators only, and the selection criteria of the adaptive threshold for various lighting conditions is not addressed. Some researchers have also suggested the use of color features for the insulator detection [14,15], which is only applicable to glass insulators.

Edge-based feature extractors were proposed in [16,21] to detect porcelain insulators from images taken from unmanned aerial vehicles (UAVs) and high-voltage live-line cleaning robots (HLCRs), respectively. Neither of the two studies presented sufficient experimental results to support the robustness of their proposed methods against the cluttered background.

Zhao et al. [22] proposed an insulator lattice model by grouping the appearance similarities of the glass and porcelain insulator and subsequently performing lattice finding using the Markov Random Field (MRF) model, combined with the spatial context information to localize multiple insulators jointly. Although the proposed method is robust against the complex background, its effectiveness is guaranteed only when a group of insulators appears together in the image, which limits the application of this method. Liao and An [23] proposed a robust multiscale and multi-feature (MSMF) descriptor based on local features and spatial orders for insulator detection in aerial images. In [24], Haar-like rectangular features and AdaBoost were used for the insulator detection. They used synthesized 3D insulator images as positive samples to train the classifier and showed significant improvement in the recognition accuracy. However, both methods [23,24] can only detect insulators in long-distance low-resolution images that are inapt for further defect analysis.

Li et al. [25] used vertical profile projection curves as the insulators’ shape features, and a SVM classifier to detect them. Wang et al. [26] proposed a Gabor features and SVM-based insulator detection method. Both shape features [25,26] rely upon the repeating pattern on the insulator, which is well observed only when the insulator images are taken at near frontal viewpoint. Consequently, insulator images with out-of-plane rotation remain unhandled by these methods. Li et al. [2] proposed to use local and global saliency map for segmenting insulators. However, their method only works when the texture and intensity of the background and the foreground regions are distinctive. This condition normally occurs only when aerial images of insulators are taken from a closer proximity.

Even though there are several proposed systems for automatic insulator detection from aerial images, the high price, and low stability make them less practical. Moreover, aerial vehicles are more susceptible to ill weather conditions (e.g., strong winds). Therefore, the ground vehicle-based systems were proposed for insulator detection [16,27,28,29] and subsequent defect analysis. Li et al. [16] used the improved MPEG-7 edge histogram descriptor (EHD) to recognize insulators from videos taken from the ground. Murthy et al. [27] computed wavelet-transform-based features and used a SVM classifier for the analysis of insulator condition. In their other work [28], they extracted features by applying a wavelet transform and subsequently utilized a hidden Markov model to segregate the damaged insulators from good ones. However, the insulator detection mechanisms in both studies [27,28] were not clearly described. Moreover, the pole detection method in [28] is based on simple edge template matching, which only works well when the poles are located against a clear background.

Oberweger et al. [30] presented a novel approach to detect insulators in aerial images based on discriminative training of local gradient-based descriptors and a RANSAC-based voting scheme. However, their scheme cannot detect multiple insulators from a single image. Jabid and Uddin [29] used a classical sliding window-based detection method with local directional pattern (LDP) feature and SVM classifier. Their method not only scales the input image to multiple sizes but also rotates the input image at multiple orientations in order to address the size and rotation variations, which slows down the detection process.

Developing a successful insulator monitoring system is a challenging problem due to the large variations in the appearances of insulators caused by scale, viewpoint, color and occlusion [22]. Cluttered backgrounds also increase the complexity of the problem and often increase the computational cost and decrease the detection rate. Most of the existing insulator detection methods address only a subset of the variations without having the capability of handling all of them. In contrast, features extracted from pre-trained CNN such as OverFeat have been successfully used in computer vision tasks such as scene recognition, object attribute detection and achieves improved results compared to handcrafted features [31]. This is the major motivation of using CNN for the detection of electrical equipment in our work. Given the power of CNN features, our work aims to automatically select the best features for various types of equipment in the CNN training routine.

In this paper, we propose a complete real-time electrical equipment monitoring system which can detect various types of insulators from live video, taken from a dual camera system mounted on the rooftop of a ground vehicle, and subsequently analyze the images for potential defects. Since most of the electrical equipment discussed in this paper are insulators, we will use the term insulator to represent all other electrical equipment throughout this paper. The camera system contains two cameras inside its casing: (1) a low-resolution (LR) camera that takes a long shot image covering many objects of interests (i.e., insulators), and (2) a high-resolution (HR) camera that focus on to these objects of interest by panning, tilting and zooming. In order to assist the camera system to detect insulators in low-resolution and high-resolution images, we trained multiple CNN-based insulator detectors. To the best of our knowledge, there has been no study where CNN was effectively used to detect and classify different types of powerline insulators (reference [32] used CNN for defect analysis, not insulator detection). The CNN-based insulator detector first detects many insulators from the low-resolution images by reading frames from the memory of the LR-camera and then passes the locations of those insulators to the HR-camera. The HR-camera then zooms into those locations and takes high-resolution images of the insulators. Another CNN-based insulator detector helps to accurately crop the high-resolution images of the insulator and passes those images to the defect analysis module. The proposed insulator detection and defect analysis system is highly robust against the cluttered background, occlusion, arbitrary orientations, diverse lighting conditions, and viewpoint changes.

To contrast the proposed scheme, the pros and cons of the previous work are summarized in Table 1. We list the number of electrical equipment that can be detected, variations and amount of complexity in the background of the images that can be handled, and the major drawbacks inherited by the previous work.

One of the most salient features of the proposed scheme is the ability to detect 17 different types of electrical equipment. Moreover, most of the existing methods present naïve approaches of thresholding the color or intensity image using a single threshold [1,14,15,16,17,25,26,33,34,35,36], and hence these methods are sensitive to color and lighting variations. In order to show the robustness of the our proposed method, we evaluated our method on a large dataset of 644 cluttered insulator images, while the methods described by [17,22,24,26,33,36] used small datasets of 2, 3, 4, 5, 10, and 74 images, respectively, and the methods in [2,21] tested their algorithm on a single image. Although [23] used 100 images for training their model, they tested their system on the same 100 images, hence the robustness of these algorithms cannot be guaranteed.

Lastly, by looking at Table 1, we can observe that only the proposed scheme provides robustness against all four types of variations, while the schemes in [29,30,37] guarantee partial robustness against out-of-plane rotation. Out of these four, the proposed scheme is the one that provides the highest precision and recall. Moreover, the scheme in [30] cannot detect multiple insulators in one image, while the proposed method can detect 17 different types of insulators in a single image. The scheme in [29] can only detect one type of insulator, and also suffers from low detection speed as discussed earlier. The scheme in [37] requires a camera with LED illuminators to illuminate extra light on the insulators so that they can be easily distinguished from the background. Moreover, their setup is only feasible for railway systems, where the distance between camera and insulators is small as compared with the distance between ground vehicle and powerline insulators.

After the detailed review of the related work and introduction of the proposed method, we highlight the main contributions of this study as the following:

(1) Unlike earlier studies that use handcraft features, we explore the robustness of the CNN features and use them for the task of multi-type insulator detection in a highly cluttered environment.
(2) We present an ellipse detection method specifically designed for segmenting the caps from various types of insulators.
(3) We propose a novel insulator rotation normalization method that normalizes the in-plane rotation of insulators, irrespective of their types.
(4) We propose a novel defect analysis method that can detect gunshot defect in polymer insulators.
(5) We present a complete automatic real-time multi-type insulator detection and defect analysis system.

The rest of this paper is organized as follows: in Section 2, we provide a detailed overview of the proposed system and discuss the multi-type insulator detection mechanism and the experimental setups used to train those detectors. We also present a novel rotation normalization method for various types of insulators, whereas in Section 3 we describe the proposed defect analysis system and its components, along with the ellipse detector algorithm. With the comparison to the state-of-the-arts in Section 4, we provide the experimental results and performance analysis of the complete insulator detection and defect analysis system. Finally, we draw the conclusion of our study with future directions in Section 5.

## 2. Overview of the Proposed System

**Figure 1:**
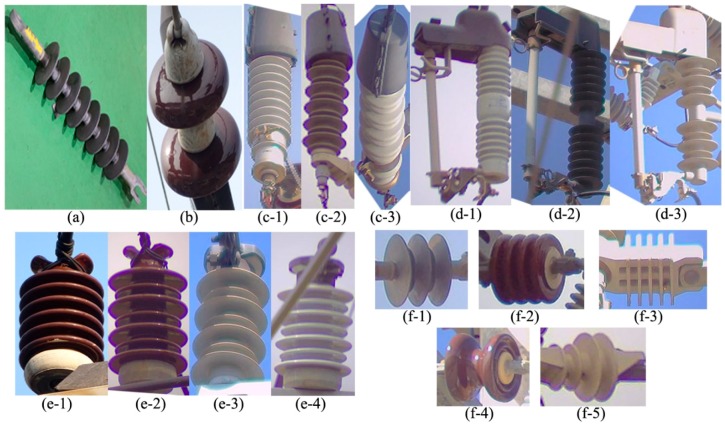
Example images of various types of electrical equipment that can be detected by our proposed system. Insulators of (**a**) polymer and (**b**) porcelain; Lightning-Arrester (LA) of (**c-1**) porcelain with uniform cap sizes, (**c-2**) polymer, (**c-3**) porcelain with non-uniform cap sizes; Cut-out-Switches (COS) of (**d-1**) porcelain, (**d-2**) polymer with uniform cap sizes, (**d-3**) polymer with non-uniform cap sizes; Line-Post (LP) of (**e-1**) porcelain with uniform cap sizes, (**e-2**) porcelain with non-uniform cap sizes, (**e-3**) polymer, (**e-4**) porcelain of white color; and COS or LA’s add-ons of (**f-1**) polymer, (**f-2**) porcelain with four caps, (**f-3**) square shaped, (**f-4**) porcelain with two caps and (**f-5**) polymer with two caps. For training CNN, we grouped the equipment into 6 base classes, i.e., (**a**) polymer insulator, (**b**) porcelain insulator, (**c**) LA, (**d**) COS, (**e**) LP, and (**f**) add-on.

This section presents a detailed overview of the proposed system. A brief introduction to the types of insulators our system can detect is given in Figure 1. In every insulator type, the repeating circular shaped part is called the “Cap” and the rod passing through the center of these caps is called the “Core” or “Sheath.” As shown in Figure 1, the proposed system can detect 17 different types of electrical equipment, having different shapes, sizes, and colors. Depending upon the availability of image data, the proposed system can be trained to detect more equipment with minor modifications in the training and detection routines.

**Figure 2:**
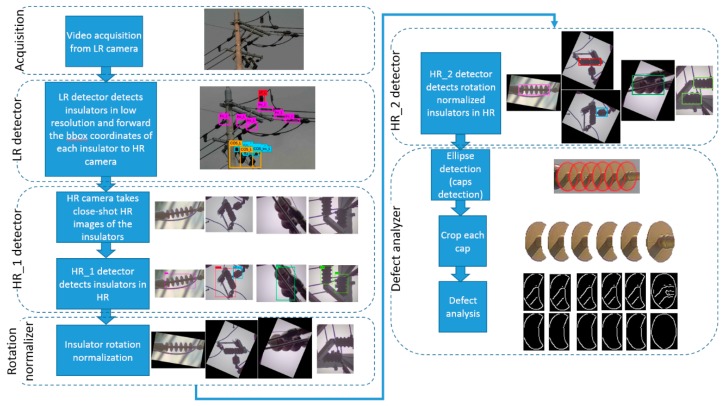
Overall system diagram of the proposed CNN-based electrical equipment inspection system.

The overall system diagram of the proposed real-time multi-type insulator detection and defect analysis system is shown in Figure 2. In the first stage, the video acquisition module (ground vehicle with the proposed camera system mounted on its rooftop) captures the video of the top part of the poles (where the insulators are installed) on the streets. This video is captured by the low-resolution camera. In the LR detection stage, the frames of this video are processed by a CNN-based rotation invariant multi-type insulator detector. As the insulator images captured by the fixed camera are of low-resolution, hence we refer to this detector as LR (low-resolution) detector throughout this paper. The LR detector passes the coordinates of the bounding boxes of the detected insulators to the HR-camera module, which takes the high-resolution close shot of all the insulators. As the vehicle is continuously moving, and the camera system has some delay in taking pictures, the HR-camera covers some extra area around the detected insulator to compensate for vehicle movement and capturing delay. For defect analysis, we need a tighter window around the insulators, so we pass the high-resolution images to another CNN-based rotation-invariant multi-type insulator detector named HR_1 (high-resolution) detector which provides more precise bounding box around the insulator body, as shown in Figure 2. The detected insulators can appear in any arbitrary orientation, hence in order to normalize the rotation of the insulators, we apply a rotation normalization method, which rotates (all types of) insulators such that the line passing through the core/shed of the insulator becomes parallel to the horizontal axis.

The rotated high-resolution images still contain extra space around the insulators, therefore, another CNN-based multi-type insulator detector named HR_2 is applied to the rotated high-resolution images, which finally give the precise bounding boxes around different types of insulators, as shown in Figure 2. The tightly cropped insulator is passed to the ellipse detector for segmenting each cap from the insulator, as shown in Figure 2. Finally, the cropped caps are passed to the defect analysis module which computes the percentage of defects in each cap. In the later subsections, we give the implementation details of the various steps shown in the system diagram of Figure 2.

### 2.1. CNN-Based Robust Insulator Detector

In contrast with the handcraft features used in the previous studies, the proposed system uses CNN for the detection of different types of powerline insulators. We believe that the lack of availability of the annotated insulator images is one of the reasons why CNN was not used in previous studies. In our work, we acquired and annotated a large and diverse set of insulator images (i.e., both, long-shot low-resolution and close-shot high-resolution images) to train the CNN-based multi-type insulator detectors.

We used Darknet’s open source neural network framework and object detection system, You Look Only Once (YOLO) version 2 [39], which is the state-of-the-art, real-time object detection method, written in C and CUDA (Compute Unified Device Architecture) programming languages. The high detection speed of YOLO reaches 50–60 frames per second on NVIDIA GeForce 1080 GPU (NVIDIA Corporation, Santa Clara, CA, USA), which makes it a suitable candidate to develop real-time applications. The high detection speed comes from the fact that YOLO divides the whole image into fixed sized regions and predicts bounding boxes and probabilities or confidence scores for each region, rather than generating the region proposals first and then applying the detection network to those regions separately [40]. As described earlier, the proposed system contains three CNN-based multi-type insulator detectors, LR, HR_1, and HR_2. In the following subsections, we explain the training and detection processes of each detector.

#### 2.1.1. Rotation Invariant Multi-Type LR Insulator Detector

**Figure 3:**
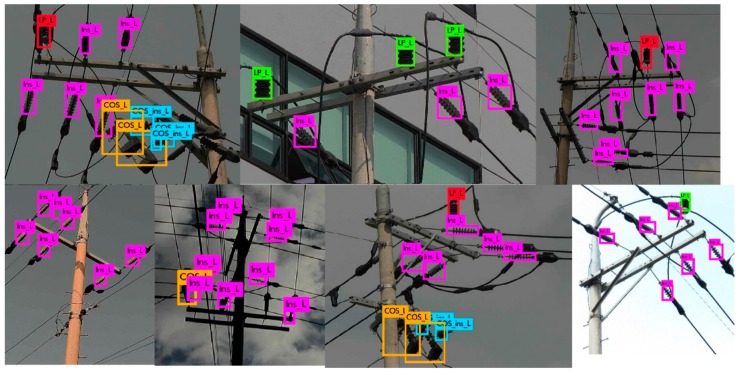
Detection results generated by our rotation invariant multi-type LR insulator detector in long-shot low-resolution images. (here symbols Ins_L, COS_L, COS_ins_L and LP represent polymer insulator, Cut-out-Switch, COS add-on and Line Post, respectively).

The LR detector is trained to detect different types of insulators from long shot low-resolution images taken by the LR camera. The detector divides the entire image into fixed sized B × B regions to detect objects. We clustered the ground truth bounding box sizes into 5 groups and set the size of B = 7 based on the average size of the bounding boxes in the major clusters. Different types of COS insulators (i.e., porcelain type, polymer type, etc.) appear very similar in the long shot images of low-resolution, as different types of LP, LA and add-on insulators. Moreover, the class information is ignored by the next step in the pipeline (as shown in Figure 2), because the class information is only used at the defect analysis step. Therefore, we combined the sub-classes of COS (Figure 1(d-1)–(d-3)), LA (Figure 1(c-1)–(c-3)), LP (Figure 1(e-1)–(e-4)) and add-on (Figure 1(f-1)–(f-5)) into their base classes, respectively. Finally, LR detector is trained with 6 classes, i.e., polymer insulator, porcelain insulator, COS, LA, LP, and add-on, denoted by Ins_L, C_Ins_L, COS_L, LA_L, LP_L, and COS_Ins_L, respectively in Figure 3. We divide the entire image set into training and test sets. We kept a steady learning rate of 0.001 throughout the 45,000 training epochs. Figure 3 shows some of the example detection results in long-shot low-resolution images.

It can be seen in Figure 3, the CNN-based rotation invariant multi-type LR insulator detector is robust against various viewing angles, scales, aspect ratios, partial occlusions, lighting variations, and cluttered environment. The background becomes more complex as we deal with close-shot high-resolution images due to the presence of other types of insulators in the background, which is discussed next.

#### 2.1.2. Rotation Invariant Multi-Type HR_1 Insulator Detector

**Figure 4:**
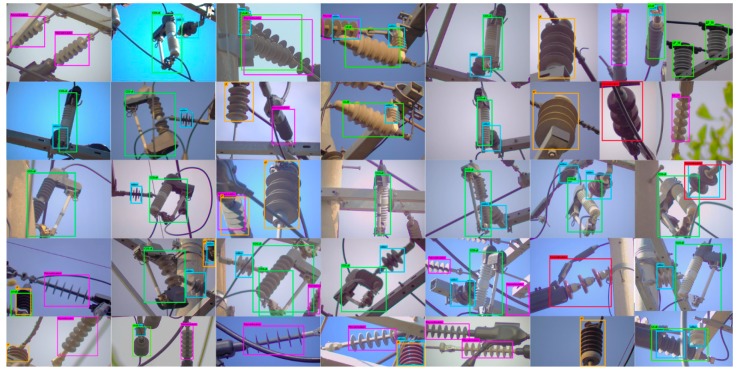
Different electrical equipment detection results by our CNN-based rotation invariant HR_1 detector. These detection results show that the used CNN-framework is very good at detecting different shapes in the cluttered environment under partial occlusion, and it is invariant against view-point, shape, size, color and lighting variations.

The image resolutions of the insulators detected by the LR detector is not sufficiently high to be used for defect analysis, hence the bounding boxes detected by the LR detector are passed to the HR camera module, which in turn guides the HR camera to pan and/or tilt and/or zoom in order to take high-resolution images of different types of insulators. Although HR_1 detector is trained to detect all the 17 different types of insulators shown in Figure 1, we combine the detection information (i.e., class labels) of each insulator into their base class (similar to what we did for training the LR detector) because the class information is ignored by the next step in the pipeline (see Figure 2). The HR_1 detector is learned with a faster learning rate for the first half of the network training process so that the network can quickly learn to distinguish between different types of insulators. In the second half of the training process, the learning rate is slowed down by a factor of 10 in order for the network to slowly learn the details of the shape, color, and context of the different types of insulators. The HR_1 detector is also robust against lighting conditions, viewpoint variations, partial occlusion, rotation, insulator sizes, and cluttered background, which is evident from the example detection results shown in Figure 4.

#### 2.1.3. Multi-Type HR_2 Insulator Detector

**Figure 5:**
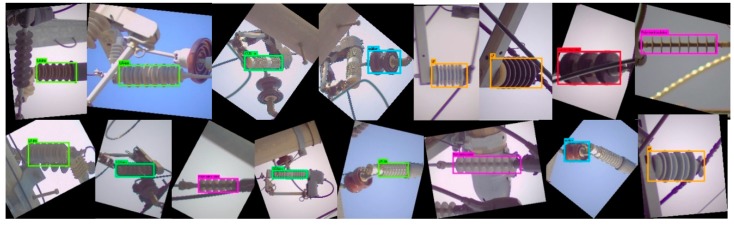
Detection results of the CNN-based multi-type HR_2 detector. The orientation of the insulator image is normalized before feeding it to the HR_2 detector. The tight and precise detection of the insulators helps in better cap segmentation by our novel ellipse detector, which is a robust alternative to the color-based segmentation [26].

As can be seen in Figure 4, the bounding boxes around the detected insulators do not tightly enclose the insulators because the insulators appear at arbitrary orientations. The proposed ellipse detection method (see Figure 2) requires that; (a) the insulator bodies must lie horizontal and, (b) the bounding boxes around the insulators’ bodies are as tight as possible for accurate ellipse detection. Hence, we apply a novel rotation normalization method (covered in Section 2.2), capable of estimating and normalizing the in-plane rotations of insulators of all types. Once the in-plane rotations of insulators are normalized, the HR_2 detector detects the bounding boxes that tightly enclose insulators. In order to train HR_2, we use rotation normalized insulator images. During annotation of the training images, we rotate and tag the bounding boxes around the insulators. The amount of rotation is used to generate the rotation normalized images for training HR_2 detector, whereas the same training images without rotation normalization are utilized for training HR_1 detector. We kept the value of parameter B = 5 for training HR_2 detector and trained the network with the same method as we used to train HR_1 detector. Each type of CNN-based detector returns a confidence score ∈[0, 1] along with the bounding box coordinates of the detected equipment. In order to reject detection results with a low confidence score, we applied a detection threshold for each type of CNN-based detectors, i.e., LR, HR_1, and HR_2. The detection results of the HR_2 detector are shown in Figure 5. The average detection time of the HR_2 detector (20 ms) is negligible, so it does not negatively affect the real-time performance of the proposed system. The novel rotation normalization approach is explained in the following subsection.

### 2.2. Insulator Rotation Normalization Method

Rotation normalization of insulator images is an essential process in our proposed system. The novel rotation normalization algorithm is designed in such a way that it can estimate the orientation of the insulators regardless of their types. We exploited the appearance symmetry property of the insulators’ shape and the spatial context information to design a robust algorithm for estimating the orientation of the insulators. The algorithm is comprised of the following steps:

(1) In order to find the best rotation angle, the edge map of the detected insulator in the high-resolution image is computed using the Canny edge detector. The edge map is rotated to all possible angles between 1 to 180 degrees.
(2) Low-level visual features of all the edge maps are analyzed exhaustively; feature points are extracted and clustered by their appearance similarities.
(3) The appearance similarity between any two feature points is computed by folding the edge map with respect to the center of the two feature points and then applying convolution.
(4) High convolution score implies that the feature points in the two folds have high appearance similarity. Hence, we do max voting on convolution scores to find candidate point cluster which is consistent with the geometrical relationship of the insulator shape.
(5) Finally, a tight bounding box encapsulating the maximum voted point cluster is returned, whose longest side represents the final orientation of the insulator.

The rotation normalized images are fed to HR_2 detector, which returns a tighter bounding box around the insulator body. The tightly cropped insulator image is then passed to the novel ellipse detector module for individual cap segmentation, which is covered in the next section.

## 3. Defect Analyzer

### 3.1. The Ellipse Detector

In order to find the defects on insulator caps, we need to analyze each and every cap separately, for which the caps of the insulator are segmented. In contrast with the color [26] based segmentation schemes which is sensitive to color and lighting variations, we propose the segmentation of the caps of the insulators using ellipse detector. Ellipse detection based cap segmentation is not only robust against color and lighting variations but also able to detect caps under major occlusion which is also unhandled by the scheme in [26]. However, traditional ellipse detection methods tend to suffer from high false positive detections due to the noisy edge map of the insulator images, and they do not take into account the appearance symmetry (same sized, equally spaced caps/sheds) prior inherited by the different insulators which can be utilized for more accurate ellipse detection. We present an ellipse detection method which takes into account the structural symmetry, number of caps, shape and other prior information for the cap detection. The overall algorithm can be broken down into three steps, i.e., (i) pre-processing the edge map and labeling the arcs, (ii) selecting proper arcs and fitting ellipse onto them, and finally, (iii) some post-processing steps to refine detection results. Even though these three basic steps are similar to the one proposed in [41,42], but we made significant changes in the underlying algorithm. The detailed steps involved in the ellipse detection algorithm are described in the following subsections.

#### 3.1.1. Adaptive Thresholding

Our ellipse detection algorithm starts with finding the edge map of the insulator image. We use the Canny Edge Detector with adaptive thresholding. In contrast with automatic thresholding used by [41,42], we formulate the adaptive threshold as a function of two terms, i.e., the total number of edges remaining in the edge map, and the minimum allowed length of the edges. Let the total number of edges in the edge map be N, $l_{i}$ be the length of the *i*th edge, $th_{el}$ be the minimum allowed edge length, $th_{canny}$ be the threshold in Canny edge detection, and $N^{\prime }$ be the number of remaining edges when an edge length threshold is applied to the edge map, then:

(1) $$N^{\prime }=\;\sum_{i=0}^{N}l_{i},\;\mathrm{where}\;l_{i}>\;th_{el}$$

**Figure 6:**
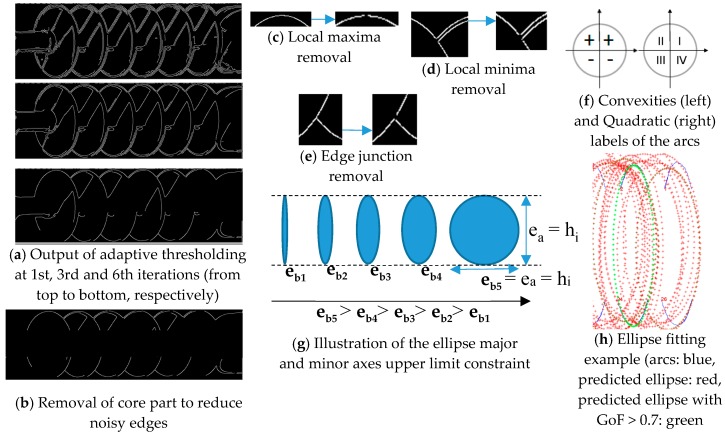
Pictorial representation of the different steps in the ellipse detection algorithm.

It is obvious that $N^{\prime }$ depends on $th_{canny}$ and $th_{el}$. We fixed the value of $th_{el}$ = 30 pixels based on the prior information of the sizes of caps in various insulators, hence $N^{\prime }$ only depends on $th_{canny}$. For ellipse detection in insulator images, we must have a minimum number of remaining edges ($N_{min}$) in the edge map, for better ellipse detection. Hence, in order to adaptively adjust the $th_{canny}$, we iteratively search the proper value of $th_{canny}$ in a gradient decent manner, until $N^{\prime }\;≅\;N_{min}$. Figure 6a shows the intermediate results of the iteration process. A different automatic thresholding method is used in [41,42], which is removing 20% of the edge pixels first, and then finding the bin index of maximum count from the histogram of edge gradients.

#### 3.1.2. Insulator Core Removal

One prior information about all the types of insulators is that a core passes through the center of the caps. The core has horizontal edges in the edge map that are sometimes connected with the edges of the caps, as shown in Figure 6a, which negatively affects the ellipse/cap detection. Hence, we remove the center part of the insulator that contains the core from edge map, which consequently removes the noisy edges at the center as shown in Figure 6b. The prior information about the sizes of insulators decides the amount of area to be removed from the center part of the edge map. Neither of [41,42] removes parts of the object for noise removal, before ellipse detection.

#### 3.1.3. Edge Refinement

Next we apply edge refinement steps to obtain arcs of good quality for robust ellipse detection. Let $e_{i}$ be the *i*th edge point in the edge map, characterized by its position and edge gradient as $e_{i}=\left(x_{i},\;\;y_{i};\;\theta_{i}\right)$. Also, let $I_{x}$,$\;I_{y}$, $I_{xy}$ represent the first order derivatives in *x*, *y*, and *xy* directions, respectively, then $D_{x}\left(e_{i}\right)=sign\left(I_{x}\right)$ represents the direction of the edge gradient. Firstly, we remove the edges that are bigger in size but their contour is flat, i.e., $D_{xy}=\left|constant\right|$. This type of edge normally shows up when insulator is partly occluded by a powerline. Secondly, we remove the edge points that lie on horizontal ($D_{y}=0$) or vertical ($D_{x}=0$) edge gradients. Thirdly, we remove the edge points that form a local minima, and local maxima as shown in Figure 6c,d, respectively. Finally, we remove the edge points that forms a junction between three or more edges, as shown in Figure 6e. Edge refinement method given in [41,42] only removes smaller edges and edges forming horizontal and/or vertical lines.

#### 3.1.4. Arc Labeling

Next, we compute the convexities and directions of all the arcs according to [41], and label all the arcs with the quadrants they belong to, based on the values of their convexities and edge gradients directions. Let $C:\alpha^{k}\to \left(+,-\right),\;\mathrm{then}\;C\left(\alpha^{k}\right)=+$ denotes that the *k*th arc represented by $\alpha^{k}$ is upper convex, and vice versa. Let the quadratic labels are represented by $Q^{I}$, $Q^{II}$, $Q^{III}$ and $Q^{IV}$, and consider the edge gradient directions are computed in an anti-clock wise manner, then the quadratic labels are assigned using Equation (2) as shown in Figure 6f:

(2) $$Q\left(\alpha^{k}\right)=\;\{\begin{array}{c} Q^{I}if\;\left(C\left(\alpha^{k}\right),\;D_{y}\left(\alpha^{k}\right)\right)=\left(+,\;+\right)\\ Q^{II}if\;\left(C\left(\alpha^{k}\right),\;D_{y}\left(\alpha^{k}\right)\right)=\left(+,\;-\right)\\ Q^{III}if\;\left(C\left(\alpha^{k}\right),\;D_{y}\left(\alpha^{k}\right)\right)=\left(-,\;-\right)\\ Q^{IV}if\;\left(C\left(\alpha^{k}\right),\;D_{y}\left(\alpha^{k}\right)\right)=\left(-,\;+\right) \end{array}$$

where Q is a function that maps $\alpha^{k}$ to one of the four quadrants, i.e., $Q:\;\alpha^{k}\to \left\{Q^{I},\;Q^{II},\;Q^{III},\;Q^{IV}\right\}$.

#### 3.1.5. Arc Triplet Selection-Constraint 1

It is clear from Figure 6f that an ellipse can be formed if and only if we combine the arcs having non-overlapping quadratic labels. Let us define the arc triplet as $\zeta^{abc}$ = ($\alpha^{a}$, $\alpha^{b}$, $\alpha^{c}$), then the arc triple $\zeta^{abc}$ is selected, if and only if, $\left(\alpha^{a},\alpha^{b},\alpha^{c}\right)$ maps to one of the triplets of $\left(Q^{I},\;Q^{II},\;Q^{III}\right)$, $\left(Q^{I},\;Q^{II},\;Q^{IV}\right)$, $\left(Q^{I},\;Q^{III},\;Q^{IV}\right)$, or $\left(Q^{II},Q^{III},Q^{IV}\right)$.

#### 3.1.6. Arc Triplet Selection-Constraint 2

The tightly cropped insulator image returned by the HR_2 detector helps in the implementation of the second constraint which allows the selection of the arc triplet, present within a possible geometrical proximity. As shown in Figure 6g, depending upon the viewpoint of the image, value of the length of the minor axis of the caps can take values in a certain range, which gives an upper limit of the possible geometrical proximity of the arcs triplet. As the methods in [41,42] are proposed for ellipse detection in general cases, they do not consider constraint 2 as in our case.

Let $e_{a}$ and $e_{b}$ denote the lengths of major and minor axes of an ellipse. Due to the tight cropping on insulator image by the HR_2 detector, $h_{i}=e_{a}$, where $h_{i}$ represents the height of the image. Also the maximum possible $e_{b}$, $e_{b}^{max}$, is equal to $h_{i}$ or $e_{a}$. Let $P_{a}\left(x_{a},\;y_{a}\right)$, $P_{b}\left(x_{b},\;y_{b}\right)$ and $P_{c}\left(x_{c},\;y_{c}\right)$ be the center points of the arcs $\alpha^{a}$, $\alpha^{b}$ and $\alpha^{c}$, respectively, then according to the second constraint, we select the candidate arc triplet $\zeta^{abc}$ if and only if, it satisfies one of the following conditions:

(i) $\zeta^{abc}\;=\;\left(Q^{I},\;Q^{II},\;Q^{III}\right)$ if $\left(\left(x_{a}-x_{b}\right)<h_{i}\wedge \left(x_{a}-\;x_{c}\right)<\;h_{i}\right)$
(ii) $\zeta^{abc}=\left(Q^{I},\;Q^{II},Q^{IV}\right)$ if $\left(\left(x_{a}-x_{b}\right)<h_{i}\wedge \left(x_{c}-\;x_{b}\right)<\;h_{i}\right)$
(iii) $\zeta^{abc}=\;\left(Q^{II},\;Q^{III},\;Q^{IV}\right)$ if $\left(\left(x_{c}-x_{a}\right)<h_{i}\;\wedge \left(x_{c}-\;x_{b}\right)<\;h_{i}\right)$
(iv) $\zeta^{abc}\;\in \;\left(Q^{I},\;Q^{III},\;Q^{IV}\right)$ if $\left(\left(x_{a}-x_{b}\right)<h_{i}\;\wedge \left(x_{c}-\;x_{b}\right)<\;h_{i}\right)$

The two constraints imposed by the algorithm not only enable better arc selection but also cut down tremendous processing time.

#### 3.1.7. Ellipse Parameter Estimation and Candidate Ellipse Selection 

Next, we fit an ellipse with direct least square fitting of ellipses method [43] using a set of points P from the three selected arcs. Let us represent a general conic by a second order polynomial:

(3) $$F\left(\propto ,x\right)=\propto .x=ax^{2}+bxy+cy^{2}+dx+ey+f=0$$

where $\propto \;=\;{\left[a\;b\;c\;d\;e\;f\right]}^{T}$ and $x={\left[x^{2}\;xy\;y^{2}\;x\;y\;1\right]}^{T}$. $F\left(\propto ;x_{i}\right)$ is called the algebraic distance of a point (x,y) to the conic F(∝,x)= 0. The fitting of the general conic may be realized by minimizing the sum of the squared distances of the curve to the M data points, $x_{i}$.

Once the proposed algorithm selects arc triplet, we pick five equally spaced points from each of the three arcs. Let $S_{p}$ and $T_{P}$ be the sampled and true points of an ellipse respectively, and $S_{p}\subseteq \;T_{P}$, where p=15 (selected points) and P is the total number of points in all the three arcs, and compute an ellipse using Equation (3). Readers are encouraged to refer [43] for more details about solving Equation (3).

If the set $S_{p}$ does not form a single ellipse or includes non-arc parts (e.g., noisy edges), the ellipse will not fit all the points $T_{P}$ on the three arcs as shown with red markers in Figure 6h. This gives us a final constraint to validate the ellipse. In order to find the overlap between the predicted ellipse *E* and the points $T_{P}$, we compute the goodness-of-fit. Let $T_{i}^{n}$ be the *i*th point on *n*th arc triple, $E^{o}$ represent the *o*th predicted ellipse, then we define a function $f\left(T_{i}^{n},\;E^{o}\right)$ that returns a point $\left(x_{i},y_{i}\right)$ on the predicted ellipse $E^{o}$, that has the minimum distance from $T_{i}^{n}$ such that the Euclidian distance $\|f\left(T_{i}^{n},\;E^{o}\right),T_{i}^{n}\;{\|}_{2}\leq \;th_{dist}$. The goodness-of-fit can be computed as:

(4) $$GoF=\;\frac{\sum_{i=1}^{P}\|f\left(T_{i}^{n},\;E^{o}\right),T_{i}^{n}\;{\|}_{2}}{P}$$

We empirically set $th_{dist}=5$ pixels. We reject the ellipse if $GoF\leq th_{GoF}$, where $th_{GoF}$ is the threshold for goodness-of-fit. We fixed the value of $th_{GoF}=$ 0.7. This step is repeated for all the candidate arc triplets.

#### 3.1.8. Combining Multiple Detections

Next, we combine multiple detections by computing the similarity between the ellipses, as:

(5) $$E_{combined}=\;Avg\left(\;F\left(\propto_{n1},x\right),\;F\left(\propto_{n2},x\right)\right),\;\;\;\;\;\;if\;\eta =1,$$

where Avg denotes averaging operation, and:

(6) $$\eta =\left(\left|e_{a}^{n1}-e_{a}^{n2}\right|\leq th_{e_{a}}\right)\wedge \left(\left|e_{b}^{n1}-e_{b}^{n2}\right|\leq th_{e_{b}}\right)\wedge \left(\left|e_{center}^{n1}-e_{center}^{n2}\right|\leq th_{center}\right)$$

η represents the measure of similarity between candidate ellipses $n_{1}$ and $n_{2},$ and $th_{e_{a}}$, $th_{e_{b}}$ and $th_{center}$ represent the thresholds for difference between; the length of major axis $\left(e_{a}\right)$, length of minor axis $\left(e_{b}\right)$ and centers of the two ellipses $\left(e_{center}\right)$, respectively.

#### 3.1.9. Clustering Detected Ellipses

The final step again exploits the symmetric structure of insulators. As same sized caps are regularly spaced to form an insulator, we cluster the candidate ellipses into a number of groups based on their similarity intervals. The cluster with the largest number of candidate ellipses is selected as the final cluster, and candidate ellipses in the cluster are considered as the detected ellipses. Again, the methods in [41,42] do not cluster the ellipses based on their physical arrangement.

**Figure 7:**
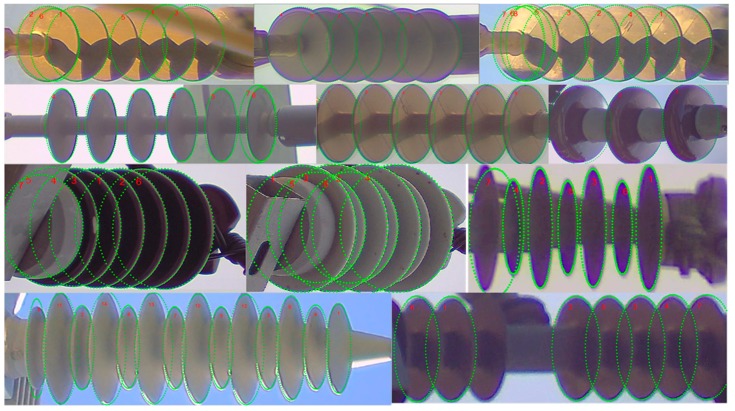
Results of the ellipse detection on different types of insulators. Green ellipses are the detected boundaries of the shed/caps. The ellipse detector makes use of repeating pattern in the insulator body and consequently less influenced by the noisy edges that are caused by shadow and overlapping shed/caps.

In Figure 7, we show some of the example ellipse detection results that prove the robustness of the proposed ellipse detection algorithm against the color and lighting variations, viewpoint changes, shadows, occlusion, and cluttered environment. The detected caps are segmented and then passed to the defect analyzer, which is presented next.

### 3.2. The Novel Bullet Shot Defect Analyzer

Defects in powerline insulators can occur due to inferior design, use of low quality materials in manufacturing, improper manufacturing processes, misapplication of the insulator or extreme stresses from weather (e.g., rain, storms, snow, hails, humidity, extreme cold or hot temperature, UV rays, etc.), vandalism, wildlife, extreme electrical activity or mishandling. These defects can alter the appearances of insulators, such, as color, shape and texture. The proposed defect analyzer provides quantitative measures of defects with insulators. Since we use optical images for the defect analysis, we only address the defects that alter the appearance of the insulators. To be more specific, those defects occurring internally without changing the outer appearance are out of the scope of this paper.

**Figure 8:**
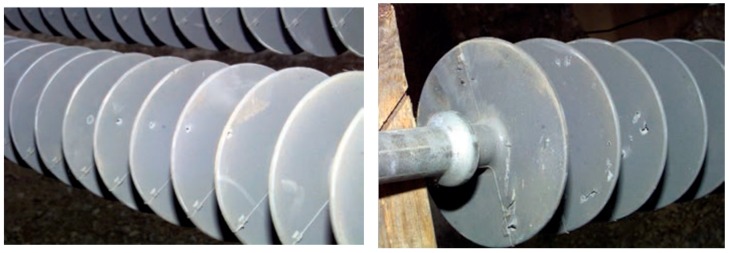
Example images of polymer insulators having bullet shot defect [45]. When hit by bullet shots, polymer insulators do not shatter like ceramic insulators because polymer is not brittle.

**Figure 9:**
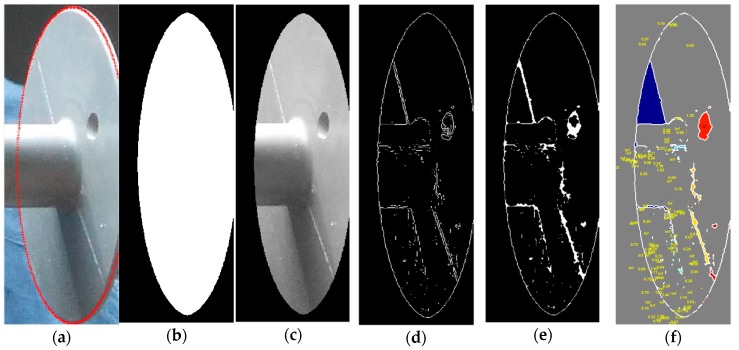
Proposed gunshot defect analysis algorithm. (**a**) Ellipse detection, (**b**) cap mask generated using the detected ellipse, (**c**) masked cap, (**d**) edge map of the masked cap, (**e**) morphologically enhanced edge map, (**f**) final detection result. A detected bullet shot marked with red color, while the numerical values in yellow color represent circularity values, in (**f**).

Figure 8 shows example images of insulators with the bullet shot defects. As the name suggests, this type of defect is caused when an insulator is hit by a bullet due to aerial gunshots. This defect reduces the electrical properties of the insulator, may lead to flashovers. Reduced electrical strengths and concerns for potential reduction of the mechanical strength are justifications for insulator replacement [44]. With the help of Figure 9, we illustrate the proposed bullet shot defect analyzer.

The defect analyzer performs the following steps to detect gunshot damage:

(1) Ellipse detection to segment caps from insulators.
(2) Masking to remove the extra background.
(3) Edge detection followed by some morphological operations to remove noisy edges.
(4) Labeling all the edges inside the cap.
(5) Connected component analysis for blob detection.
(6) The bullet shots normally create circular shaped holes on insulator caps. Hence, in order to differentiate between noisy edges and edges caused by gunshots, we compute the circularity [46] of every closed contour as:
  (7) circularity=ρ= 4πAcontourΨ2
  where $A_{contour}$ and Ψ are the area and perimeter of a closed contour, respectively. The circularity of a perfect circle is equal to 1. The perimeter Ψ is a scalar quantity defined as the distance around the boundary of the contour, computed by summing together the positive distance between each adjoining pair of pixels around the border of the contour. If there are total *I* points on the curve, then Ψ can be computed as:
  (8) $$\Psi =\overset{I-1}{\underset{i=1}{\sum }}\sqrt{{\left(x_{i+1}-\;x_{i}\right)}^{2}+\;{\left(y_{i+1}-\;y_{i}\right)}^{2}}$$
(7) A priori information related to the color intensity of the hole created by bullet shot puts a final constraint in classifying the detected contour as bullet shot or not. We observed that in every insulator image with bullet shot damage, the color intensity of the area inside the hole of bullet shot is lower than the surroundings.
(8) Let $\mu_{hole}^{I}$ and $\mu_{boundary}^{I}$ represent the mean intensity of the hole and boundary pixels, respectively, then the final detected contour is classified as a bullet shot if:
  (9) $$\left(\rho \geq th_{\rho }\right)\wedge \left(A_{contour}/A_{cap}\geq th_{Area}\right)\wedge (\mu_{hole}^{I}<\mu_{boundary}^{I})$$
  where $th_{Area}$ represents the minimum ratio of the area of bullet shot hole divided by the size of the cap in pixels, and $th_{\rho }$ denotes the minimum value of circularity of the detected hole to be considered as a bullet shot defect.
(9) If there are total *G* contours classified as gunshot damage, then the final percentage of damage per cap due to the gunshot is computed as:
  (10) Dgunshot= ∑i=1GAcontouriAcap
  where $A_{contour}^{i}$ represents the area of *i*th gunshot contour.

## 4. Experimental Results and Discussion

In this section, we present experimental results of the proposed insulator detection and defect analyzer system.

### 4.1. Database Acquisition

There is no publicly available insulator image dataset and only few studies presented quantitative evaluation result of the system performance using self-acquired small databases. In our work, we gathered a large, unconstrained dataset of insulator images and presented our evaluation results based on that. We believe that the dataset is unbiased, unconstrained and sufficiently large enough to validate the reliability and effectiveness of the proposed system. We acquired a large dataset of long-shot low resolution and close-shot high-resolution images of equipment. Among them, we manually annotated 667 low resolution and 5533 high-resolution images (6200 images, in total). Detailed numbers of each insulator types used for training and test are provided in Table 2.

### 4.2. Insulator Detection Results

We used the well-known Pascal Score [47] to evaluate the performances of the three CNN-based multi-type insulator detectors. The Pascal score is calculated by taking the Intersection-over-Union (IoU) of the detected bounding box $BB_{detected}$ and the ground-truth bounding box $BB_{gt}$ as:

(11) $$IoU=\;P\left(BB_{detected},\;BB_{gt}\right)=\frac{Area\left(BB_{detected}\cap \;BB_{gt}\right)}{Area\left(BB_{detected}\cup \;BB_{gt}\right)}$$

According to the criteria in [47], an object is considered correctly detected if $P\left(BB_{detected},\;BB_{gt}\right)\geq th_{IoU}$ where $th_{IoU}$ is typically set as 0.5. In the following subsections, we present the detection accuracies of the different CNN-based detectors and the proposed rotation normalizer.

#### 4.2.1. Rotation Invariant Multi-Type Low Resolution Insulator Detector (LR)

For long-shot low-resolution images, our objective is to detect as many insulators as possible. We keep the detection threshold lower (= 0.10) to allow some false positive detections because the false positives are removed at later stages by the high-resolution detectors. Moreover, even if the detection returns a wrong classification label, the detected insulator has a high chance to be correctly classified by the high-resolution classifiers. Since the high-resolution classifiers are trained with high-resolution images, they learn a better shape model as compared with low-resolution classifiers. We found that most of the false positives detected by LR detector are corrected by HR_1 detector, and only 0.001% of the false positives detected by LR detector remain after HR_2 detector. We used 70% of images of each type of equipment for the training and the rest for the test.

Table 2 summarizes the detection performance of the CNN-based low-resolution (LR) detector. As can be seen in Table 2, the LR detector performs best for polymer insulator. The performances of other types of equipment appear relatively lower, possibly due to the smaller number of training samples. As LR detector is trained with low-resolution images, it requires more training data to develop a better shape model for different types of the insulators.

Moreover, we found that the $P\left(BB_{detected},\;BB_{gt}\right)>0.5$ criteria is very strict for the LR detector as the trained CNN model struggles with the localization of smaller objects in low-resolution images [40]. Considering the aforementioned aspects, the results in Table 2 can be considered as a promising towards the ultimate goal.

#### 4.2.2. Rotation Invariant Multi-Type High-Resolution Insulator Detector 1 (HR_1)

The purpose of rotation invariant HR_1 detector is to detect insulators in the close-shot high-resolution images taken by the camera module. Similar to LR detector, we keep the detection threshold low (= 0.30) to allow more detections. As our rotation normalization method utilizes symmetry property of the shape of insulators, the false positives that contain asymmetric shapes, such as trees, pole edges, wires, insulator covers, etc. can also be suppressed in the rotation normalization step. We verified that 95% of the false positives detected by HR_1 detector are suppressed by our rotation normalization step, while out of the all false positives detected by HR_1 detector, none is passed to the defect analyzer.

Table 2 also summarizes the statistics of the detection results of the HR_1 detector. As compared with the performance of LR detector, the HR_1 performs much better even with the smaller number of training samples. We believe that this is due to the fact that HR_1 detector is trained with high-resolution images, and hence it learns the shape of the insulators in more detail compared to the LR detector.

#### 4.2.3. Multi-Type High-Resolution Insulator Detector 2 (HR_2)

The final CNN-based detector is responsible to detect insulators in rotation normalized images. The performance of HR_2 detector is summarized in Table 2. Training images used to train HR_1 detector are reutilized to train HR_2 detector, with their orientations are normalized. The average performance of HR_2 detector is slightly lower than the average performance of HR_1 detector due to the reason that Pascal criterion is very strict for rotated bounding boxes, which is not originally intended [47]. As can be observed from Figure 5, the HR_2 detector returns much tighter bounding boxes (intended to be used in post-processing steps) which sometimes fails to fulfill the $P\left(BB_{detected},\;BB_{gt}\right)>0.5$ criteria. Regardless of this limitation, average precision and recall values of the HR_2 detector are sufficiently high enough to be used in the insulator inspection system.

In order to find the accuracy of the rotation normalizer, we used the ground truth orientation information saved during the annotation process of close-short high-resolution images. We allow a tolerance of ±5° degrees, under which we consider the estimated orientation as correct estimation. We evaluated our insulator normalizer on all the samples shown in Table 2 and found an average accuracy of 88.20%. The average time to compute the rotation is 18.56 milliseconds.

### 4.3. Comparison with the State-of-the-Art

Unavailability of publicly available dataset restricts the comparison of our results with those of state-of-the-art methods. Among the published studies, only [29,30] presented their detection performance on polymer insulator using the same standard metric as used in this paper, i.e., Pascal score. The Precision-Recall curve (PR curve) given in [29,30] represents the detection evaluation of only one type of insulator, i.e., polymer insulator, hence for the fair comparison, we also compute the PR curve by evaluating the proposed CNN-based detectors on polymer insulator only.

As mentioned in Section 3, all three types of CNN-based detectors return confidence values ∈[0, 1] along with the bounding box coordinates of the detected insulator, hence we generate the precision and recall values of the three types of detectors by sliding a threshold over all confidence values to obtain different true positives/false negative rates. In addition, we also compared the performance of the published insulator detector algorithms (wherever reported in terms of precision and recall) with the proposed CNN-based detectors and summarized them in Table 3.

**Figure 10:**
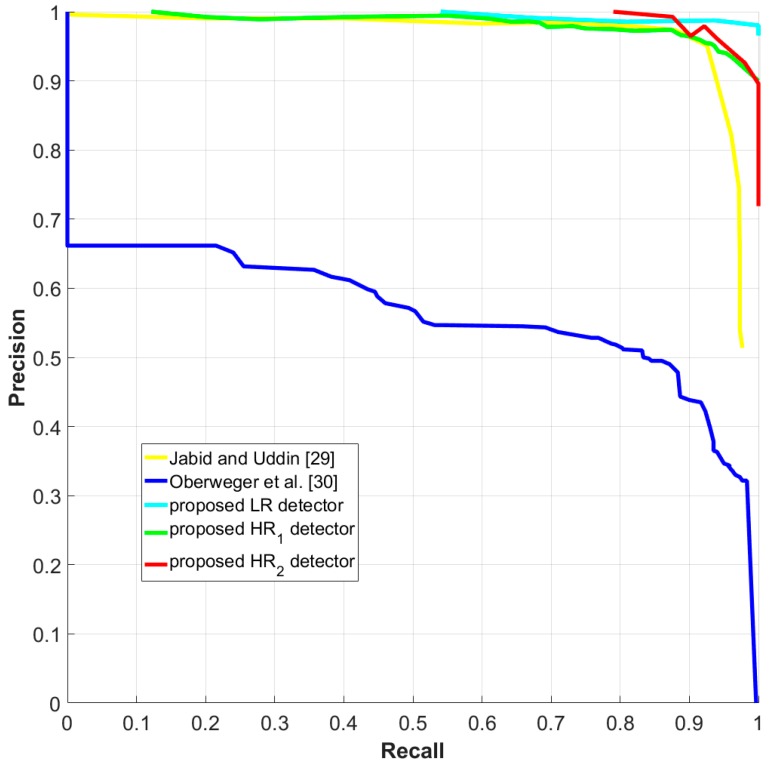
The precision-recall curve for insulator detection. The comparison is drawn between Uddin [29], Oberweger et al. [30], and the three types of CNN-based insulator detectors proposed in this paper.

The PR curve shown in Figure 10 and the performance statistics in Table 3 clearly depicts the superior performance of all the three proposed CNN-based insulator detectors. Although the size of the evaluation dataset we used (high-resolution images) is bigger than the evaluation datasets used by [3,23,29,30], yet the robust CNN-based detectors outperform the handcraft features used in [3,23,29,30].

### 4.4. Defect Analyzer Results

We present the performance of the defect analyzer in Table 4. The evaluation dataset consists of 70 polymer insulator images, including four images of defective insulators. After ellipse detection, 547 caps were retrieved including 24 caps with bullet shot defects. Table 4 shows the values of precision and recall at different values of $th_{Area}$ and $th_{\rho }$.

The two thresholds also prove to be a strong geometrical constraint in the detection of true bullet holes and significant rejection the false positives (noisy edges), because, usually the bullet shots leave a circular hole in caps, and the size of that hole is relatively small as compared with the size of the cap. If bullet shots break/tear off the major portion of the cap, then we consider this case as a separate defect, called broken shed/cap, which is not covered in this manuscript. As shown in Table 4, reducing the values of these thresholds allow more detections at the cost of reduced precision. In general, a high recall value is desired in the defect analyzing systems designed for electrical safety to prevent electric failure. Subsequent visual verification can be carried out to remove false alarms manually. In either case, the sensitivity of the defect analyzer can be tweaked with the help of the two thresholds, as mentioned in Table 4. Table 4 shows that the proposed bullet shot defect analyzer is robust against noisy edges due to shadows, notches on caps surface, diverse illumination conditions and improper cap detections.

### 4.5. Discussion about the Results and Challenges of the Proposed System

Even though, the proposed CNN-based electrical equipment inspection system present state-of-the-art detection performances, it contains room for improvement. Hence in the following subsections, we discuss the presented results along with the challenges and limitations faced by the proposed system.

#### 4.5.1. Availability of Annotated Data

The CNN-based detection frameworks usually (and other machine learning framework in general) require a large amount of training data to properly learn the convolution filters. We observed an overall performance improvement of 3% in precision and 0.7% in recall when using 50% of the augmented data (by horizontally flipping the images) [48] in our experiments. The amount of required train data also depends upon the complexity of the problem, e.g., the shape, color, and size of the object to detect, number of classes, the relationship between the classes, etc. As shown in Figure 1 and Figure 3, Figure 4, Figure 5, the powerline equipment exhibits extreme shape, color, size, aspect-ration, lighting, viewpoint variations, we feel the need to gather more annotated training and testing data to further improve the results.

#### 4.5.2. Train Data Class Imbalance

The potential cause of the less precision and recall values for the ceramic insulator, LP, COS, LA and add-on in Table 2 is the fact that there is a high class imbalance between the number of samples of polymer insulators and other equipment. In fact, polymer insulator images are about 82% of the total training data, and hence during the training process, when the training optimizer computes the overall training loss, it appears to be very small because the validation process can easily achieve 82% accuracy by simply predicting polymer insulators. And that is one of the reasons why the precision and recall of the polymer insulator is better than the precision and recall of the other equipment. We believe that if we train the network with more training samples for equipment other than polymer insulators, or build a cost-sensitive loss function, we can achieve better overall detection results.

#### 4.5.3. Training Time

As the CNN-framework used in this paper does not contain a region proposal network (RPN, [40], p. 6518), rather it tries to find objects in the fixed sized grid, so its detection time is very fast as compared to the CNN-frameworks containing RPN, while it takes a longer time to learn the possible size and positions of the objects in the dataset, hence the training time of our CNN-based detector is more than the training time of RPN based network. Our CNN-based detector takes an average of 48 h to train, but shows a detection time of 0.023 s per frame (~43 frames per seconds), while the network with RPN roughly takes 16 h to train, but it offers a detection time of seven frames per second [40] (p. 6521), which is far from meeting the needs of a real-time inspection system.

#### 4.5.4. Valid False Positives

**Figure 11:**
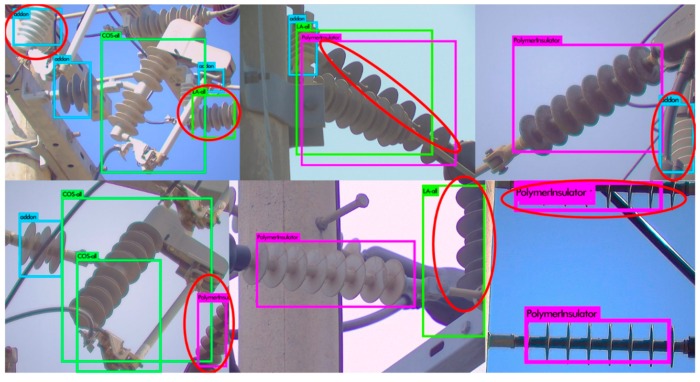
Robustness of CNN-based electrical equipment detector leads to the detection of occluded equipment, which is treated as false positive by our testing routine. Examples of these false positive detections are marked with red circles in this figure.

Our training routine automatically extracts negative regions from the input image by considering the region other than the ground truth bounding box as negative. In order not to detect the equipment whose body is occluded more than 50% (because highly occluded equipment cannot be effectively tested for defects), during the annotation process, we marked such occluded equipment as “ignore” (not used as positive or negative samples during the training process). But the trained CNN network is highly robust against occlusion, and it still detects occluded objects with high confidence. Although, these detections are valid (i.e., detects the right object), our testing routine consider these detections as false positives, and hence reduce the overall reported performance of the system. Figure 11 shows some of these detection results.

#### 4.5.5. Limitations in the Ellipse Detection Algorithm

As discussed earlier, our ellipse detector algorithm requires that the rotation of the input insulator image be normalized, and the insulator body tightly cropped, due to which we use a rotation normalizer and HR_2 detector. Hence, in the proposed system, there is a trade-off between the accurate ellipse detection and the computational resources used by the additional rotation normalizer and the HR_2 detector.

**Figure 12:**
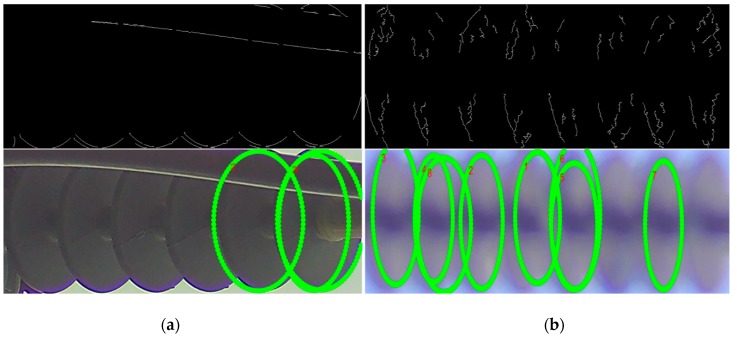
Robustness of ellipse detector depends upon the edges response. The figure shows two ellipse detection results along with their input edge responses. (**a**) The edges of the caps are occluded by the wire passing in front of the insulator, (**b**) while the blurry image resulted in a noisy edge map, and subsequent noisy detection results.

Furthermore, even though the proposed ellipse detector is robust against noisy edges, still the ellipse detector algorithm relies on the quality of edges. As the ground vehicle taking the videos of the electrical equipment is continuously moving, there are cases where the detected electrical equipment is blurred out or occluded in such a way that their important edges are not retrieved. In such cases, the ellipse detector struggles to detect the caps, as shown with some example cases in Figure 12.

#### 4.5.6. Availability of Defected Cap Images

Images of caps damaged by gunshots or any other damage are scarce and due to this reason, our testing set is highly imbalanced (95% of the segmented caps have no gunshot defect). Consequently, the precision drops more drastically as we try to improve the recall. A 100% precision and 75% recall shown by the gunshot defect analyzer means that the system is able to reject 100% false positives in 523 healthy caps, at the cost of rejecting only six faulty caps as false negatives, which is one false negative per 87 images (still a quite good ratio).

## 5. Conclusions and Future Directions

In this paper, a novel automatic electrical equipment detect and inspection system has been proposed. The proposed system can detect 17 different types of electric power line equipment from videos taken by a ground vehicle and analyze one type of defects in polymer insulators. Compared to previous methods, the proposed system is superior in many aspects: (a) powerful CNN-based insulator detection versus handcrafted features, (b) capability to detect 17 types of insulators, (c) use of close-shot high-resolution images for defect analysis, enabling more precise defect analysis of insulators, (d) evaluation of detection performance on a large diverse dataset of long-shot (204 test samples) and close-shot images (807 test samples), which proves the robustness of the proposed system against occlusion, lighting conditions, in-and-out of plane rotations, viewpoint changes, color, shape, texture and cluttered environment.

The proposed insulator rotation normalization and ellipse detection methods enable the system to recognize cap level defects. In order to support real-time operations, the proposed system is accelerated by a GTX1080 GPU (NVIDIA Corporation, Santa Clara, CA, USA) and uses OpenCV, CUDA and cuDNN libraries along with Darknet’s CNN framework. The proposed system is tested on a large, unbiased, unconstrained evaluation dataset of insulator images and state-of-the-art results are presented. Comparisons with previous studies verified the superior performance of the proposed system.

The system pipeline presented in this work provides a natural guide to future research, which includes pushing existing CNN models to learn different types of insulators at deeper levels and formalizing for detection of additional types of insulator defects. The future research will also consider studying a specific and unified CNN architecture for insulator detection and insulator defect analysis.

## Figures and Tables

**Table 1:** Summary of the related work.

Method	Published Year	# of Equip. that Can Be Detected	Can Handle Variations in				Background Type	Major Drawback
			Color	Lighting	Shape	View-Point		
[2]	‘17	1					△	Saliency only works when the main object is close to the camera. Single image used for training and test which cannot prove the robustness of the system
[14,15]	’11	1					△	High dependency upon color pixel values
[16]	‘10	1					⬤	Simple template matching based approach
[17]	‘10	1					△	Works only when there is low intensity bg and high intensity fg
[25]	‘12	1					⌘	Works with close shot images only. Whole technique depends upon naïve approach of binarizing the intensity image.
[33]	‘18	2					⌘	Naïve color thresholding
[35]	‘17	1					△	Color based detection is severely affected by lighting variations. Assumption of structural symmetry fails when view-point-changes.
[36]	‘15	1					⌘	Whole technique depends upon naïve binarizing the intensity image.
[26]	‘16	1			✓		△	Whole technique depends upon color thresholding. Single image for testing and training which cannot prove the robustness of the system.
[21]	‘06	1	✓				⌘	Single image is used to train and test, so robustness of the scheme cannot be proved
[38]	‘15	1	✓				△	Active contour model only works when the main object is very close to camera and bg color is discriminant with respect to fg. Testing dataset contains only 12 images.
[22]	‘12	2	✓	✓			⌘	Algorithm completely depends upon structural symmetry, which is lost when viewing angle changes, Detection results are shown on only two images
[34]	‘18	1			✓	✓	△	Saliency only works when main object is close to the camera and bg is far from the main object. Color and structural feature are effected by lighting and view point changes
[23]	‘15	5	✓	✓	✓		⌘	Cannot handle out-of-plane rotation
[24]	‘14	1	✓	✓	✓		⬤	Low precision and recall, Small testing data (only 10 images)
[27,28]		1					⬤	Segmentation algorithm is not given so the results are implausible
[30]	‘14	1	✓	✓	✓	Par.	⌘	Cannot detect multiple instances of insulator in an image, precision is also very low
[29]	‘17	1	✓	✓	✓	Par.	⌘	Sliding window based detection, as well as detection at multiple rotation angles limits the real-time processing
[37]	‘18	1	✓	✓	✓	Par.	⬤	Only applicable to rod-insulator in railway catenary system. System require special type of camera module. Precision is very low, almost 1 false positive per image.
Our		17	✓	✓	✓	✓	⌘	Require GPU for real-time processing. Takes 3~4 days for training with large dataset. Require two cameras.

fg = foreground, bg = background, ⬤ = simple background, △ = complex but discriminative background, ⌘ = complex background, Par. = Partially handle.

**Table 2:** Summary of performances of the proposed LR, HR_1 and HR_2 detectors.

Equipment Type	LR Detector			HR_1 and HR_2 Detectors			LR Detector		HR_1 Detector		HR_2 Detector	
	#Train Samples	#Test Samples	Total #Samples	#Train Samples	#Test Samples	Total #Samples	Precision (%)	Recall (%)	Precision (%)	Recall (%)	Precision (%)	Recall (%)
*Polymer Insulator*	730	204	934	2745	644	3389	99.05	89.94	98.11	92.69	96.94	93.76
*Porcelain Insulator*	64	20	84	50	13	63	11.63	29.41	92.31	97.30	79.49	81.58
*COS*	132	23	155	148	39	187	49.21	68.89	85.54	100.00	88.73	85.14
*LP*	117	45	162	140	32	172	76.32	54.74	75.86	93.62	50.46	96.49
*LA*	127	19	146	65	26	91	29.03	36	60.00	94.74	86.49	72.73
*Add-on*	112	28	140	208	53	261	60.00	58.82	98.88	87.13	76.19	48.98
*Total #Samples*	**1282**	**339**	**1621**	**3356**	**807**	**4163**						
*Avg.*							**77.08**	**77.37**	**92.14**	**92.98**	**88.39**	**87.18**

**Table 3:** Summary of performance comparison (Polymer Insulator Only).

Method	Evaluation Dataset Size	Recall (%)	Precision (%)
Jabid [29]	500	94.24	89.54
Wu and An [3]	50	86.47	85.59
Liao and An [23]	100	91	87
Oberweger et al. [30]	375	98	33
Proposed LR	204	**99.05**	**89.94**
Proposed HR_1	644	**97.92**	**92.16**
Proposed HR_2	644	**95.91**	**91.57**

**Table 4:** Performance results of the defect analyzer.

Defect Type	Precision (%)	Recall (%)	$th_{Area}$	$th_{\rho }$
**Bullet shot defect (results at different values of $th_{Area}$ and $th_{\rho }$)**	100	75.00	0.007	0.4
	77.87	87.5	0.0045	0.3
	65.92	100	0.001	0.2
